# Severe Eosinophilic Syndrome Associated with the Use of Probiotic Supplements: A New Entity?

**DOI:** 10.1155/2012/934324

**Published:** 2012-12-06

**Authors:** Fabian A. Mendoza, Shivani Purohit, Lawrence Kenyon, Sergio A. Jimenez

**Affiliations:** ^1^Jefferson Institute of Molecular Medicine and Scleroderma Center, Thomas Jefferson University, Philadelphia, PA 19107, USA; ^2^Rheumatology Division, Department of Medicine, Thomas Jefferson University, Philadelphia, PA 19107, USA; ^3^Department of Pathology, Anatomy and Cell Biology, Thomas Jefferson University, Philadelphia, PA 19107, USA

## Abstract

Severe eosinophilic syndromes related to the administration or use of unsuspected immunogenic substances have been described previously. Many of these diseases presented initially as clusters or isolated cases. The spanish toxic oil syndrome, the eosinophilia myalgia syndrome, and nephrogenic systemic fibrosis are examples of such diseases. We describe 2 cases of a severe eosinophilic syndrome characterized by marked peripheral blood eosinophilia (>15,000 cells/ml), mononeuritis multiplex, and necrotizing vasculitis which developed in a close temporal association with the recent onset use of nonprescription probiotics. There was no history of a prior autoimmune disease. Although both cases had prompt response to immunosuppression with rapid resolution of peripheral blood eosinophilia and accompanying constitutional symptoms, they remained with permanent neurological deficits.

## 1. Introduction

Probiotics are defined by the World Health Organization/Food and Agriculture Organization (WHO/FAO) as live microorganisms that when administered at appropriate doses confer a health benefit to the host by regulation of the gastrointestinal flora [1, 2]. Most frequently, these include *Lactobacillus* or *Bifidobacterium* species as well as a range of lactic acid producing bacteria. Being considered a dietary supplement in the USA, the Food and Drug Administration (FDA) regulation of probiotics is not as strict as that for prescription drugs [3]. On the other hand, there is an increased trend in the use of over-the-counter probiotics in patients with rheumatic diseases based on the belief that their use can be beneficial for regulating the immune response beyond the gastrointestinal tract. Whereas the evidence for these claims is marginal at best, there have been numerous reports of adverse events following probiotic use including bacteremia with *Lactobacilli*, *Saccharomyces Boulardii* fungemia and endocarditis in postoperative patients, as well as *Saccharomyces Cerevisiae* fungemia in immunocompromised patients [4–6]. This report describes two patients who developed a severe eosinophilic syndrome with vasculitis and mononeuritis multiplex, temporally associated with probiotic use. Both patients recalled using a new brand of probiotic between 2 and 4 weeks prior to symptom onset. 

## 2. Case Reports

**Case 1**

**Figure 1:**
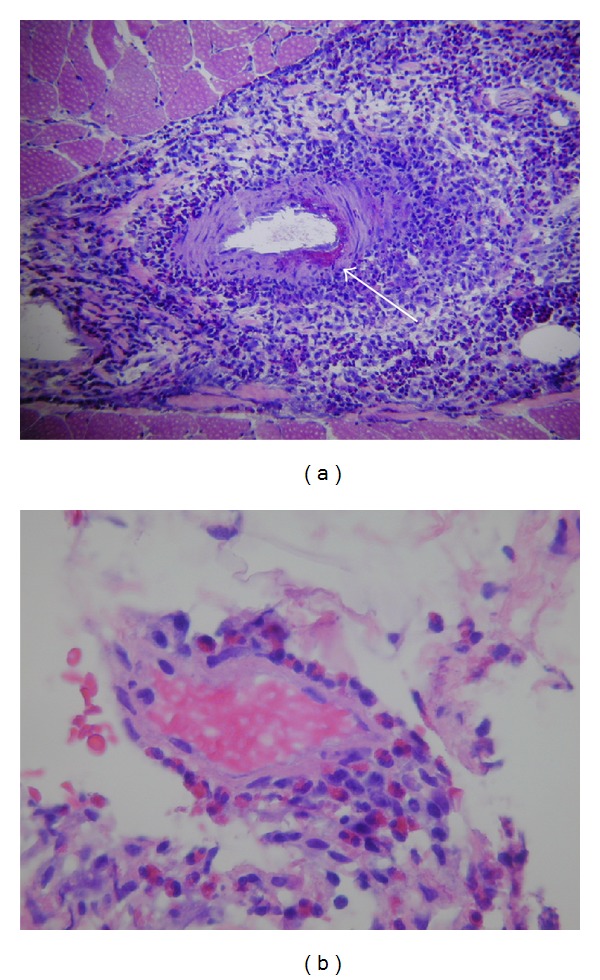
Case 1 (A and B). (a) Cryostat section of quadriceps muscle stained with hematoxylin and eosin demonstrates a severe necrotizing vasculitis of an intramuscular arteriole. Intramural fibrinoid necrosis is evident (arrow). Surrounding the vessel are large numbers of macrophages, lymphocytes, and eosinophils (original magnification 100x). (b) High-magnification image of formalin fixed, paraffin-embedded tissue with an intramuscular vessel demonstrating large numbers of intramural and perivascular eosinophils (original magnification 400x).

A 55-year-old female with no significant past medical history, presented with sudden onset of arthralgias, myalgias, malaise, night sweats, lower extremity weakness, and paresthesias of 1-week duration. Physical and neuromuscular examination revealed bilateral moderate-to-severe lower extremity weakness, asymmetrical in severity and predominantly distal with right foot drop. She was found to have leukocytosis (29,700 cells/mL) with 56% eosinophils (16,632 cells/mL). Electromyography (EMG) and nerve conduction studies revealed mononeuritis multiplex. Right quadriceps muscle and right sural nerve biopsies performed before the initiation of IV steroids or cyclophosphamide showed severe necrotizing eosinophilic vasculitis (Figure 1). The sural nerve biopsy showed secondary axonal degeneration. Examination of bone marrow demonstrated mild eosinophilia consistent with a reactive process without evidence of malignancy. Flow cytometry of peripheral blood cells was negative for phenotypically abnormal cells. She was treated with IV corticosteroid pulses (methylprednisolone 1 g IV for 3 consecutive days) followed by oral steroids (prednisone 1 mg/Kg/d) with slow titration downward over 10 months and cyclophosphamide 1000 mg/m²/month for 6 months, followed by azathioprine 2 mg/kg/d after the initial 6 months.

**Case 2**

**Figure 2:**
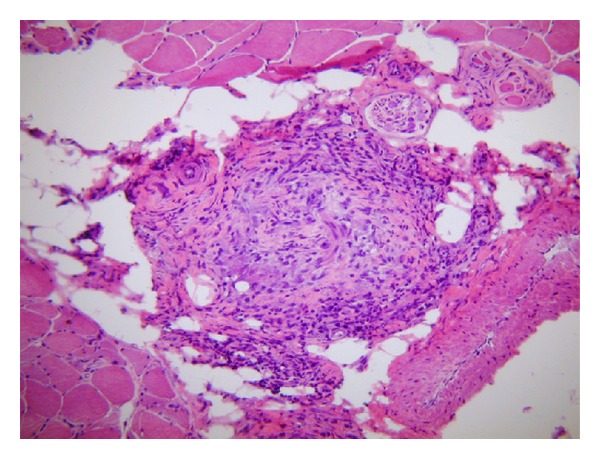
Case 2. Cryostat section of muscle stained with hematoxylin and eosin demonstrates an intramuscular arteriole with mural infiltrate of lymphocytes and histiocytes and luminal obliteration. No eosinophils were present; however, the biopsy was obtained one week after IV corticosteroid administration.

A 65-year-old female with a history of inactive mild episodic asthma, sinusitis, and a remote history of Hashimoto's thyroiditis presented with sudden onset of abdominal pain, left arm weakness, numbness, right foot drop, diplopia, and profound general malaise. Physical and neuromuscular examination revealed mild left arm weakness along with profound distal right lower extremity weakness and foot drop. No skin lesions were noticed. Laboratory studies showed leukocytosis of 24,300 cells/mL with severe eosinophilia of 76% (18,468 cells/mL). EMG and nerve conduction studies showed evidence of mononeuritis multiplex and in addition, a mild underlying diffuse peripheral neuropathy. A right quadriceps muscle biopsy performed 1 week after initiation of IV steroids showed necrotizing vasculitis without eosinophils (Figure 2). By the time the biopsy was performed, the peripheral blood eosinophilia had resolved and apparently also the eosinophilic component of the vasculitis. A right sural nerve biopsy performed at the same time demonstrated a moderate chronic axonal neuropathy with numerous degenerating myelinated and unmyelinated fibers but no evidence of vasculitis. Bone marrow studies demonstrated 45% eosinophils without evidence of malignancy or atypia. She was treated with the same initial therapeutic regimen as Case 1 which included IV corticosteroids and oral cyclophosphamide, followed by mycophenolate mofetil (MMF) 2 grams/day.

Extensive questioning did not disclose the use of any other over-the-counter products, including L-tryptophan, recent use of leukotriene inhibitors, or any other new medication. However, both patients recalled the use of a new brand of probiotics described as an “extra-strength concentration” in a boxed blister pack, purchased over-the-counter in a Philadelphia metropolitan area pharmacy. Unfortunately we were unable to unequivocally identify the specific brand. None of the patients had gastrointestinal complaints, but they used the probiotic to “improve their immune system”.

Both patients were negative for FIP-1 (PDGF) mutations which are commonly present in myeloproliferative forms of eosinophilic syndrome. Furthermore, there were no abnormalities detected by peripheral blood or bone marrow flow cytometry, nor abnormal PDGF-related translocations or other cytogenetic abnormalities. None of the patients developed respiratory symptoms or had pulmonary infiltrates on high-resolution chest-computed tomography scans.

Response to IV corticosteroids followed by immunosuppressive therapy was optimal in both cases, with rapid resolution of peripheral blood eosinophilia and of the generalized symptoms. Although there was some degree of neurological improvement, to date, both patients have remained with permanent motor and sensory deficits in the lower extremities requiring orthotic devices for ambulation.

## 3. Discussion

 We describe here the clinical manifestations and extensive laboratory, histopathologic, and radiological studies of two patients who developed an abrupt onset of a severe eosinophilic syndrome with prominent constitutional symptoms, necrotizing vasculitis, and mononeuritis multiplex, occurring in close temporal association with the ingestion of a probiotic supplement. Both of the patients were initially diagnosed with Churg-Strauss vasculitis, and one of them fulfilled the American College of Rheumatology diagnostic criteria proposed for Churg-Strauss [7]. There was histological evidence of an eosinophilic necrotizing vasculitis in one of the cases, whereas the second case showed vasculitis without tissue eosinophils. It is very likely that the absence of eosinophils in the tissue vasculitic infiltrate in the second case is related to the IV corticosteroid therapy which was instituted prior to the biopsy. However, a more detailed analysis taking into consideration the severity and sudden onset of their general symptoms, absence of prior history of asthma in one of the patients (and nonactive mild episodic asthma in the other), absence of lung parenchymal involvement in both patients, negative ANCA (immunofluorescence and ELISA MPO/PR3 antibodies), marked eosinophilia (above 50% of the total WBC in both patients), and the close temporal association with consumption of a probiotic agent leads us to conclude that their disease was induced by the ingestion of a foreign agent presenting as an eosinophilic syndrome. Thus, we believe that these two cases may represent a novel eosinophilic clinical entity distinct from Churg-Strauss syndrome. 

 The cases presented differ from eosinophilia myalgia syndrome (EMS) because of the absence of skin edema or fibrosis, the lack of a history of L-tryptophan ingestion, and the presence of acute neurological involvement as opposed to the subacute or chronic course of neurological manifestations typically described in EMS [8]. 

 Numerous severe eosinophilic syndromes related to unsuspected immunogenic substances have been well described in the past presenting initially as either clusters or isolated cases which subsequently reached epidemic proportions. The Spanish toxic oil and EMS are good examples [8, 9]. Although it was generally accepted that the EMS epidemic had disappeared following the withdrawal of the contaminated L-tryptophan containing preparations, a nonepidemic case of EMS has very recently been reported [10]. In both disorders, the initial presentation of isolated or clustered cases associated with either a nonregulated contaminated food or dietary supplement delayed the recognition of the etiological agent and the prevention of new cases [11]. Both of the patients described here presented to medical attention in the same month, and apparently both acquired the suspected agent at approximately the same time raising epidemiological concerns of additional unreported or undiagnosed cases.

 Prior experience in unveiling the etiology of the eosinophilic syndromes has shown that a high index of suspicion is required. The description of these two cases may allow the recognition of similar cases and remind the medical community that the widespread use of agents seemingly considered to be beneficial and free of side effects may occasionally have serious consequences. In this paper, we welcome government efforts focused on the establishment and enforcement of regulations promoting “good manufacturing practices” for dietary supplements.
